# Fault Detection, Isolation, Identification and Recovery (FDIIR) Methods for Automotive Perception Sensors Including a Detailed Literature Survey for Lidar

**DOI:** 10.3390/s20133662

**Published:** 2020-06-30

**Authors:** Thomas Goelles, Birgit Schlager, Stefan Muckenhuber

**Affiliations:** VIRTUAL VEHICLE Research GmbH, Inffeldgasse 21a, 8010 Graz, Austria; Birgit.Schlager@v2c2.at (B.S.); Stefan.Muckenhuber@v2c2.at (S.M.)

**Keywords:** automotive, perception sensor, lidar, fault detection, fault isolation, fault identification, fault recovery, fault diagnosis, fault detection and isolation (FDIR)

## Abstract

Perception sensors such as camera, radar, and lidar have gained considerable popularity in the automotive industry in recent years. In order to reach the next step towards automated driving it is necessary to implement fault diagnosis systems together with suitable mitigation solutions in automotive perception sensors. This is a crucial prerequisite, since the quality of an automated driving function strongly depends on the reliability of the perception data, especially under adverse conditions. This publication presents a systematic review on faults and suitable detection and recovery methods for automotive perception sensors and suggests a corresponding classification schema. A systematic literature analysis has been performed with focus on lidar in order to review the state-of-the-art and identify promising research opportunities. Faults related to adverse weather conditions have been studied the most, but often without providing suitable recovery methods. Issues related to sensor attachment and mechanical damage of the sensor cover were studied very little and provide opportunities for future research. Algorithms, which use the data stream of a single sensor, proofed to be a viable solution for both fault detection and recovery.

## 1. Introduction

Advancing the level of automation for vehicles is a major challenge in today’s automotive industry. Automated vehicles are expected to provide great benefits for the driver and enable new transportation use cases and applications, e.g., [1]. Improving passenger safety is among the key arguments for the development of automated vehicles. Today, more than 1.35 million people die in road traffic crashes each year, making road traffic crashes the leading cause of death among children and young adults between 5 and 29 years of age [2]. Advanced driver assistance system (ADAS) and automated driving (AD) functions have the potential to reduce this number significantly, since most car accidents are traceable to a human error [3,4,5].

The Society of Automotive Engineers (SAE) classifies six levels of driving automation [6]. Currently, available vehicles provide up to SAE level 2 “partial driving automation”, where an ADAS function can take over lateral and longitudinal vehicle motion control. Examples are Cadillac’s Super Cruise, Mercedes-Benz’s Drive Pilot, Nissan’s ProPILOT Assist, Tesla’s Autopilot, and Volvo’s Pilot Assist. Vehicles that fulfil SAE level 3 “conditional driving automation” must provide an AD function that allows the driver to remove his attention off the road and only intervene when the system requests. In this case, the vehicle is responsible for object and event detection and proper response. Going from SAE level 2 to level 3+ implies that the responsibility for environment perception is transferred from the driver to the vehicle.

Automated vehicles work according to the SENSE-PLAN-ACT cycle [1]. SENSE represents everything related to environment perception. Within PLAN the necessary decisions are made based on the information from SENSE. ACT includes the controlling of steering, acceleration, and deceleration of the vehicle based on commands from PLAN. This chain of command illustrates how important a reliable environment perception system is for every automated vehicle. In particular, SAE level 3+ vehicles, that cannot rely on a human driver monitoring the environment constantly, have very high demands in terms of robustness and reliability. A combination of diverse and redundant sensor types is required to provide a robust environment perception during all possible environmental conditions. A sensor set including camera, radar (radio detection and ranging), and lidar (light detection and ranging) sensors is considered the best option to fulfil the SENSE demands of level 3+ vehicles [7] eventually.

Sensor faults, either caused by internal malfunctions (e.g., broken antennas) or disturbing external factors (e.g., adverse weather conditions), propose a considerable danger for each automated vehicle’s anticipated functionality. This can be health and even life-threatening for passengers in the vehicle and people in the proximity of automated vehicles. A standard method to tackle sensor faults of critical systems in the aviation or military domain is sensor redundancy. However, in the automotive industry, the cost factor plays a significant role and therefore proposes a limiting factor to sensor redundancy.

**Figure 1:**
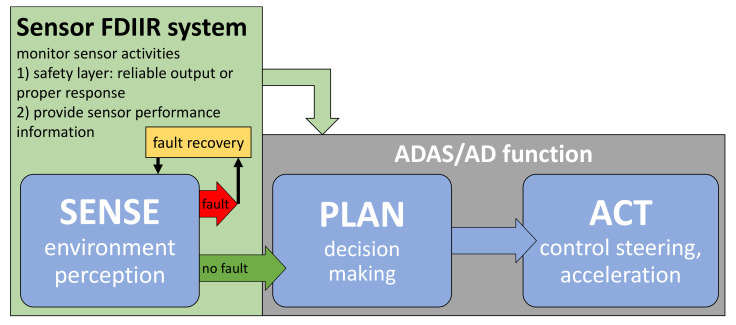
Schematic illustration of the sense-plan-act cycle including a sensor FDIIR (fault detection, isolation, identification, and recovery) system that monitors the sensor activity to ensure a reliable environment perception.

To address sensor faults in automated vehicles in a cost effective manner, sensor fault detection, isolation, identification, and recovery (FDIIR) systems, e.g., [8] can be included into each individual perception sensor that is contributing to the SENSE-PLAN-ACT cycle (Figure 1). The sensor FDIIR system constantly monitors the activity of the respective perception sensor for correct operation. In case of a detected sensor fault, the system intervenes by preventing erroneous information reaching the PLAN step, and tries to recover the affected sensor (e.g., a wiper removes dirt from the optical aperture of the sensor). Additionally, FDIIR systems can provide sensor performance information to the ADAS/AD function (e.g., adverse weather conditions are currently reducing the sensors field of view (FOV) by 50 %) to increase the information content for the PLAN step.

Two EU research projects are currently addressing faults of automotive perception sensors under unfavourable environmental conditions. The RobustSENSE project [9] aims to develop a system performance assessment module, which shall monitor the reliability of every perception sensor in the vehicle under adverse weather conditions. The DENSE project [10] approaches the problem by developing a fused sensor set that combines radar, gated short-wave infrared camera, and short-wave infrared lidar to provide a reliable perception under challenging conditions.

Sensor FDIIR systems are equally important for all types of automotive perception sensors, i.e., camera, radar, and lidar. This publication introduces a classification schema for faults, detection methods and recovery methods that applies to all perception sensor types in the automotive domain.

The literature survey and state-of-the-art analysis in this publication put the focus on FDIIR systems for lidar sensors, since lidar is a recently emerging sensor for automotive applications, and there is far less experience in the automotive industry with lidar compared to radar and camera. Today’s level 2 vehicles typically rely on radar and camera sensors to perceive the environment [7]. However, lidar sensors are expected to play an essential role in level 3+ vehicles, since lidars provide a three-dimensional depth map with very high angular resolution [11,12]. High prices of mechanically spinning lidars are currently a limiting factor, but costs will decrease significantly with new technologies such as optical phased array, MEMS-based mirrors, etc. [11,13]. For example, Druml et al. [14] introduced a lidar prototype that shall eventually lead to commercially available lidar sensors with a range of more than 200 m for costs less than USD 200.

The main objectives of the presented study are the following:

- Identify all types of faults, detection methods, and recovery methods for automotive perception sensors and develop a corresponding classification schema.
- Evaluate the state of the art of FDIIR methods for automotive lidar.
- Explain, discuss, and compare the most promising existing FDIIR methods for automotive lidar.
- Identify research opportunities related to FDIIR methods for automotive lidar.

The publication is structured as follows: Section 2 introduces a new fault classification for automotive perception sensors and relates the fault classes to current and upcoming international safety standards for automated vehicles. Section 3 introduces a corresponding classification schema for fault detection methods and Section 4 introduces a corresponding classification schema for fault recovery methods. Section 5 describes the methods applied in the literature survey on lidar FDIIR methods that is presented in Section 6. Section 7 completes the paper with a discussion and conclusion and gives an outlook on future work.

A glossary of the technical terms can be found at the end of this paper.

## 2. Classification of Faults of Perception Sensors

**Figure 2:**
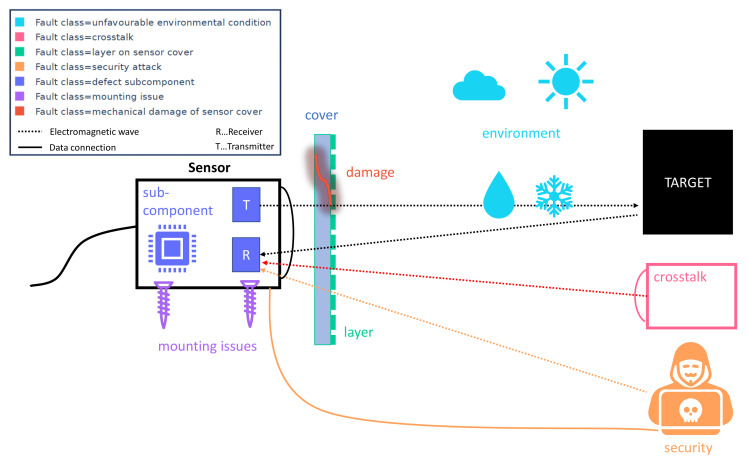
Schematic illustration of sensor fault classes.

Faults can occur on different levels in an automotive perception system. Ranging from the whole perception system, including the sensor fusion layer, to the individual sensor and its components. Faults on each level need different detection and recovery strategies. In this review, we focus on the individual sensor and its subcomponents, disregarding the perception algorithms that extract information based on raw data from individual sensors or perception systems consisting of several sensors. Therefore, we consider faults that occur until the raw data of the sensor is generated before any optional object detection and classification algorithm is applied (Figure 2).

Different faults occurring in perception sensors may have a similar cause. Therefore, perception sensor faults can be classified. A classification of faults is useful when it comes to the design of fault detection, fault isolation, fault identification, and fault recovery algorithms since similar faults may benefit from similar or equal algorithms. We suggest a classification into the following fault classes: defect subcomponent, mechanical damage to sensor cover, layer on sensor, mounting issue, security attack, unfavourable environmental condition, and crosstalk (see Figure 2). Table 1 lists the seven fault classes including exemplary faults and international safety standards addressing the faults.

A defect subcomponent is a faulty internal part of the sensor, e.g., a defect transmitter, a defect receiver, or a defect processing unit. A mechanical damage to the sensor cover may be scratches, cracks, or holes in the sensor cover but also a missing sensor cover or a deformation of the sensor cover count to this fault class. A layer on the sensor cover like dirt, water, ice, salt, or snow can cause that objects are not detected (false negatives), that not existing objects are detected (false positives) or that objects are misclassified. Mounting issues may be a change of the sensor’s position or vibrations while driving. A further risk are security attacks as shown in, e.g., [15,16]. This may be a security attack by another active electromagnetic source, i.e., denial of service and false data injection, or an electronics hack over a wired or wireless connection to the sensor. Furthermore, a perception sensor has limitations under different unfavourable environmental conditions, which can cause, e.g., a reduction of the field of view. Other active perception sensors may cause a fault in the sensor data because of crosstalk, as described, e.g., in [17,18]. The difference to security attacks with laser hacks is that crosstalk occurs accidentally, and security attacks occur by malicious hacking.

### International Safety Standards Addressing Faults of Perception Sensors

The international standard ISO 26262 [19] and the international specification ISO PAS 21448 [20] represent the state-of-the-art for safety standardization of road vehicles. FDIIR methods play an important role to fulfil the current and upcoming requirements of ISO 26262 and ISO PAS 21448 for automotive perception sensors. This section explains, how the above introduced fault classification schema (Section 2) relates to the ISO 26262 and ISO PAS 21448.

The ISO 26262 “Road vehicles—Functional safety” from 2011 includes safety measures and safety mechanisms to ensure functional safety of electrical and electronic systems of road vehicles. The standard differentiates between systematic faults and random hardware faults and provides a systematic development framework for electrical and electronic systems. The Automotive Safety Integrity Level (ASIL) is defined in this standard. This level defines which requirements and measures have to be considered and implemented to avoid an unreasonable risk [19]. Concerning FDIIR methods for automotive perception sensors, the ISO 26262 especially applies to the sensor fault class defect subcomponent listed in Table 1. This fault class affects the sensor itself and is not caused by sensor limitations due to the environment.

The ISO PAS 21448 “Road vehicles—Safety of the intended functionality” addresses limitations of systems where no fault, according to ISO 26262, occurred. The letter code PAS implies that this is a publicly available specification and not a standard at that point in time. The rework of the PAS version to an international standard has started in the beginning of 2019, and it is planned to be released as an international standard by the end of 2021. The ISO PAS 21448 especially applies to the limitations of systems that are based on sensing the environment. Such limitations can be caused by, e.g., different environmental conditions or other road users. The Annex F of the standard lists such limitations [20]. The sensor fault classes mechanical damage to sensor cover, layer on sensor cover, mounting issue, security attack, unfavourable environmental condition, and crosstalk listed in Table 1 address the issues described in the ISO PAS 21448. These sensor fault classes are caused by triggering conditions, which are combinations of sensor weaknesses in the design or specific technology or negative influencing factors of the environment.

## 3. Classification of FDII Methods for Perception Sensors

Fault detection, isolation, and identification (FDII) methods determine whether a fault occurred in a system, where the fault occurred, and how severe the fault is. The term “fault diagnosis” is often used as a synonym, e.g., in Chen and Patton [21], Chen et al. [22], Piltan and Kim [23].

The fault detection unit or fault detection algorithm of a perception sensor determines whether a fault occurred in the perception sensor or not. If the fault detection unit decides that a fault occurred, the fault isolation unit identifies where the fault occurred and which part of the sensor is faulty. When the location of the fault is determined, the fault identification unit determines the severity of the fault and the level of sensor degradation.

To categorize FDII methods for perception sensors, we suggest a classification into the following classes (Table 2): comparison to sensor model, monitoring sensor output, comparison to static ground-truth, comparison to dynamic ground-truth, comparison to other sensor of same type, comparison to other sensor of different type, monitoring internal interface, and comparison of multiple interfaces.

**Figure 3:**
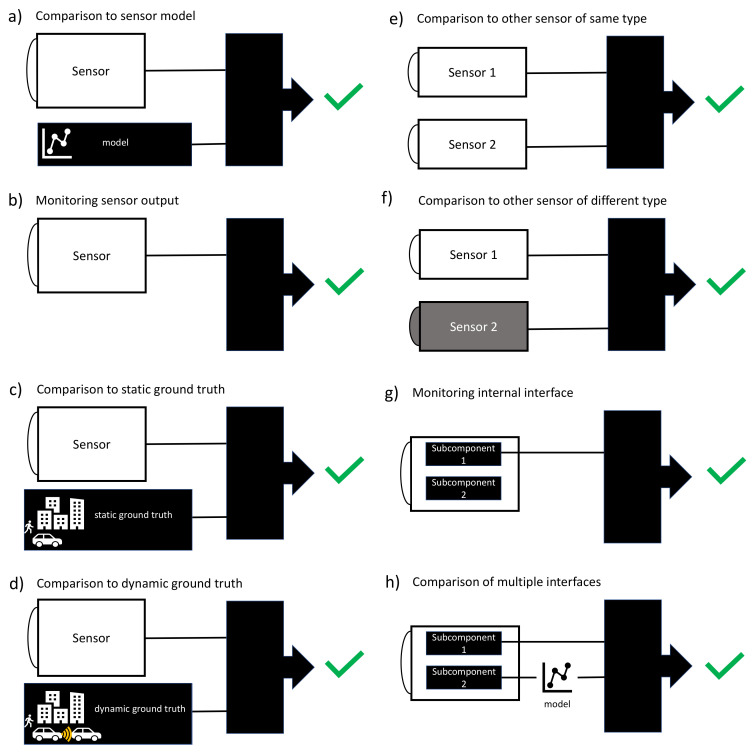
Schematic illustration of FDII methods.

The class comparison to sensor model includes the comparison of real sensor measurements with the output of a sensor model (Figure 3a). In general, comparing a process with its model is a very common method in FDII literature for simple systems like bearings. Monitoring a perception sensor with this method requires very detailed knowledge about the environment and a sensor model.

The class monitoring sensor output refers to methods that use only the output data of the sensor under observation (Figure 3b). The monitoring algorithm determines whether the sensor is healthy or faulty, the fault’s location, and the severity of the fault solely based on the sensor’s output.

The class comparison to static ground-truth identifies faults based on comparing the sensor’s output with static ground-truth data (Figure 3c). Static ground-truth may be immobile objects (houses, traffic signs, …) stored in a map or well-defined test targets placed at known locations specifically for sensor assessment.

The class comparison to dynamic ground-truth identifies faults based on comparing the sensor’s output data with dynamic ground-truth data. Dynamic ground-truth may be obtained by connected infrastructure in terms of vehicle to everything (V2X), e.g., vehicles that collect and broadcast data with their own sensors (Figure 3d). Furthermore, the position and the geometry of other vehicles may be sent from vehicle to vehicle (V2V) to check if the sensor on the ego-vehicle perceives the right position and geometry of the other vehicle.

The class comparison to other sensor of same type identifies faults based on comparing the output of two sensors of the same type, e.g., two lidar sensors, two radar sensors, or two camera sensors, with overlapping FOV (Figure 3e).

Contrary, the class comparison to other sensor of different type identifies faults based on comparing the output of two sensors of different types, e.g., lidar compared to radar, with overlapping FOV (Figure 3f). Therefore, an algorithm that transforms the data of one sensor to comparable data of the other sensor has to be applied.

The class monitoring internal interface identifies faults based on monitoring the output of a single internal sensor interface (Figure 3g). The monitoring algorithm determines whether the sensor is healthy or faulty, the location of the fault, and the severity of the fault based on the output of a single sensor interface.

The class comparison of multiple interfaces identifies faults based on comparing the output of two interfaces of one sensor (Figure 3h). The subcomponents between the two interfaces are modelled. A fault can be detected by comparing the model’s output at a specific interface with the real output of the same interface.

## 4. Classification of Recovery Methods for Perception Sensors

If the FDII system detects a fault, a fault recovery method can be applied to eliminate or mitigate the fault and restore the functionality of the perception sensor and thereby the system that relies on the sensor.

To categorize fault recovery methods for perception sensors, we suggest a classification into the following classes: software adaption, hardware adaption, temperature regulation, and cleaning of sensor cover. Table 3 shows fault recovery classes and respective exemplary methods.

Software adaption as a recovery class includes algorithms that are either applied on the sensor output data to mitigate faults directly or that are applied in the processing chain that uses the sensor data, for example, filtering of the point cloud for unwanted signals from precipitation.

Similarly to software adaption, it is also possible to apply hardware adaption, if required. Hardware adaption can be adjusting the sensor’s alignment after a change of the sensor’s position, or aligning the sensor cover if only a part of the sensor cover is faulty.

A further recovery class is temperature regulation. This can be heating if the sensor is covered by an ice layer, for example, or cooling to prevent overheating of the sensor.

The last recovery class, cleaning of sensor cover, is required in case of a disturbing layer on the sensor cover. For example, wipers may be used to remove dirt or water from the sensor cover.

## 5. Literature Survey Methodology

This section explains the methods used for the literature survey, presented in the following Section 6. Relevant publications addressing faults, fault detection methods, and recovery methods for automotive lidars were identified, analysed and then classified in a quantitative approach based on the PRISMA scheme, e.g., [24,25]. The presented literature survey methodology can also be applied for literature studies on other perception sensors.

**Figure 4:**
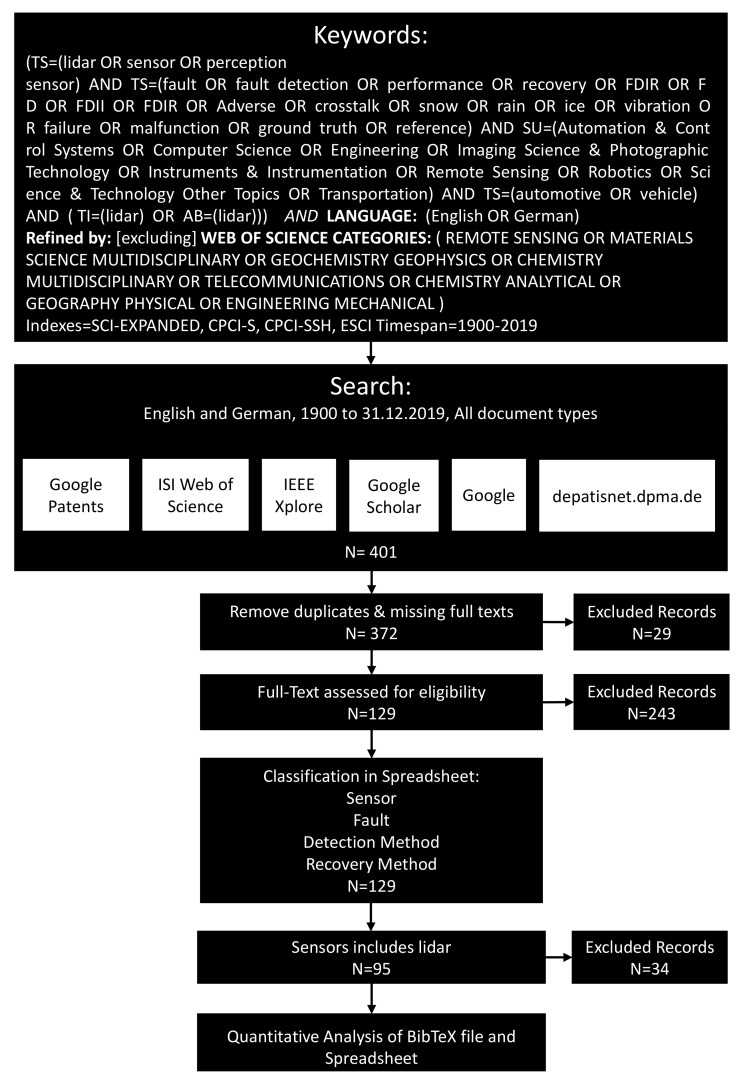
Review process for FDIIR methods of automotive lidar sensors.

Figure 4 shows the literature search procedure adopted for identifying relevant studies. We selected the interdisciplinary databases ISI Web of Science (also known as Web of Knowledge), the engineering database of IEEE, and the patent search engines of Google and depatisnet. Further, we also included a general Google search to cover grey literature and websites in addition to the research articles, conferences, and patents. The search was conducted between August and December of 2019; therefore we included sources published between 1900 and 31 December 2019.

After the initial search, we removed duplicates and missing full texts. In the next stage, we assessed the documents for eligibility. Here, we excluded records that were outside the focus of this study, i.e., records which did not mention any of the following: a fault, a detection method, or a recovery method. In terms of recovery methods, the focus was put on publications, where the sensor itself can perform the recovery method. This means that the following recovery solutions were excluded: recovery in the form of functional redundancy, an adaption of sensor fusion, direct or hardware redundancy, or fall back to a safe state like “limp home” and shut down.

In the next stage the literature was categorized according to the classification schema introduced in Table 1, Table 2 and Table 3 and the results were stored in a spreadsheet. Followed by filtering oft records which did not explicitly deal with lidar, leaving 95 records which were further analysed.

The records were stored in a BiBteX file and were analysed together with the spreadsheet file with Python. The bibliography processor Pybtex (https://pybtex.org) was used to parse the BibTeX files. The BibTeX key was used to link the BiBteX file with the spreadsheet. For further details, please refer to the supplements, which include the BibTeX file, the spreadsheet, and the saved Web of Science search queries.

## 6. Literature Survey on FDIIR Methods for Automotive Lidar

**Figure 5:**
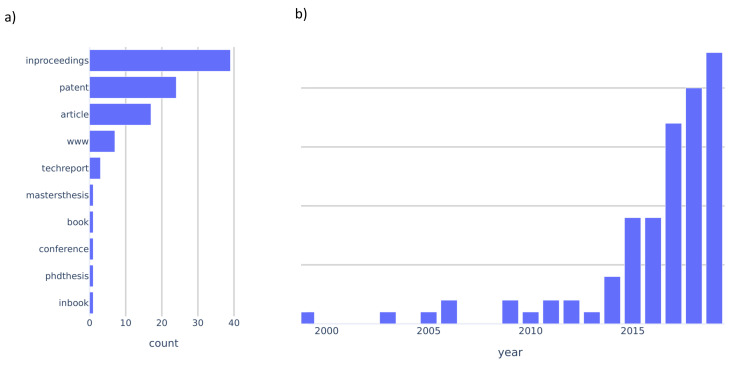
Overview of BibTeX publications which were analysed (N = 95). (**a**) shows the counts of BibTeX classes. (**b**) shows the number of publications per year.

This section provides an overview of the state-of-the-art of fault detection, isolation, identification, and recovery methods for automotive lidar and their faults. We identified and collected 95 highly relevant publications. Out of those publications the majority were conference papers, followed by patents and journal articles, as Figure 5a shows. Of those publications, the majority were published in the last five years, as can be seen in panel Figure 5b. Since 2014, there has been a steady increase in publications until 2019. This increase is most likely due to the recent focus of the automotive industry on lidar technology.

### 6.1. Fault Classes of Automotive Lidar

**Figure 6:**
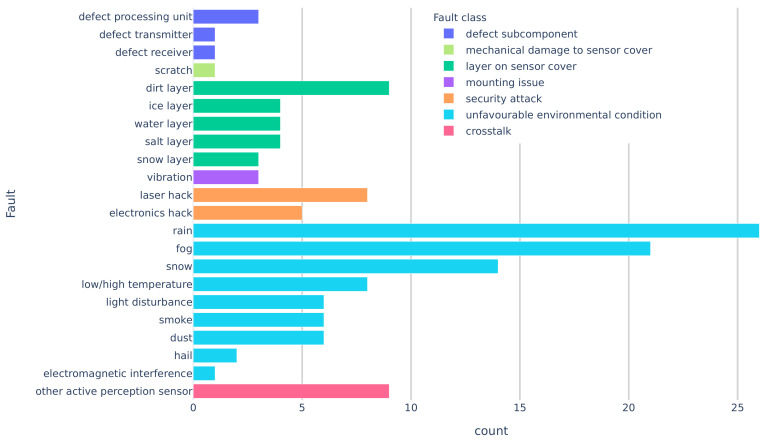
Counts of mentioned faults in the literature.

First, we classified the literature according to the fault classes. Table 4 and Figure 6 give an overview of the mentioned faults and fault classes in the literature. Faults belonging to the class unfavourable environmental condition are mentioned in 46 documents, making it the class with the most entries. Followed by layer on sensor cover and security attack. Mounting issues are covered in two documents and mechanical damage to sensor cover only in one. The total number of records is larger than the number of publications because one publication can present several fault classes.

### 6.2. FDII Classes and Realizations for Automotive Lidar

**Figure 7:**
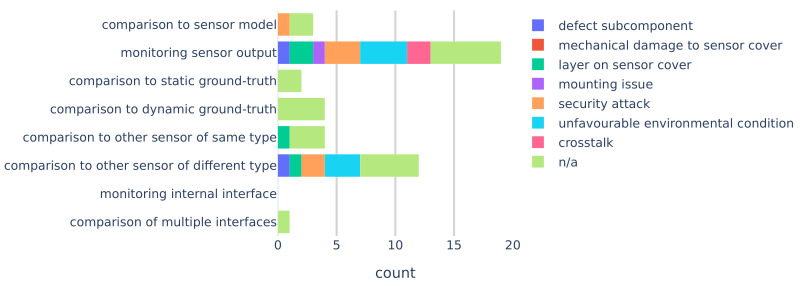
FDII classes count with colour coding according to the mentioned fault class in the publication. Some papers present fault detection methods without mentioning a specific fault, as indicated by the “n/a” category.

Figure 7 shows the number of mentions per FDII class according to the definitions in Section 3. The most common method of FDII is monitoring the output of a single sensor. Detecting faults by comparing different sensors has been described 12 times, which makes it the second most common method. FDII by monitoring internal interface was never mentioned.

In the comparison to sensor models method, residuals are calculated between the output of the sensor and a sensor model. Faults are indicated when the residual is above a threshold value. For example, a general observer scheme with extended Kalman filters is described by Huang and Su [95]. Several publications describe sensor fault detection based on monitoring sensor output with different realizations. For example, Tascione and Bode [101] detects lidar faults by checking if spin rate, laser power, laser frequency, and photodetector alignment are in its tolerance ranges. Jokela et al. [84] evaluate the standard deviation of range measurements under different environmental conditions. Some publications like Leslar et al. [99] and Segata et al. [26] describe outlier detection. Filters or machine learning approaches may be applied to sensor data to suppress outliers. For example, a supervised learning method is mentioned by Breed [98], which used pattern recognition algorithms and neural networks. Furthermore, James et al. [33] used deep neural networks to classify sensor contaminations. An alternative method used for lidar fault detection is the comparison to static ground-truth which is described in Zhu and Ferguson [105] and Bruns and Venator [104]. As described in these publications, static ground-truth may be landmarks, road markings, or terrain maps. Contrary to using static ground-truth as reference data, some publications describe how to implement the comparison to dynamic ground-truth. Dynamic ground-truth is typically received from other vehicles in the vicinity over V2V or by servers over V2X as described by Mehrdad [106] and Ghimire and Prokhorov [107]. The three patents described in Zhu et al. [108], Zhu et al. [110] and Kim et al. [109] utilize the comparison to sensor of same type. A distance function is used to decide whether an error occurred or not. For example, in Kim et al. [109] a fault is detected if the Mahalanobis distance exceeds a specified threshold. Kim et al. [109] detects the fault first and then recalibrates the sensor whereas Zhu et al. [108] and Zhu et al. [110] describe no further reactions after fault detection. Another method is the comparison to a different type of sensor, which is typically more complex, since the data format has to be transformed before comparison. Dannheim et al. [52] use a camera and a lidar to detect and classify unfavourable environmental conditions like snow, rain, and fog. Daniel et al. [62], as another example, uses a radar, a lidar, and a stereo camera under foggy conditions. Other publications like Choi et al. [46] and Guo et al. [43] describe how to suppress security attacks by comparing between sensors of different types.

### 6.3. Recovery Methods for Automotive Lidar

**Figure 8:**
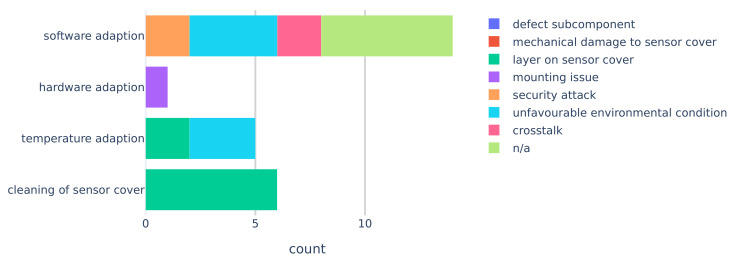
Recovery class count colour coded by fault classes.

In terms of recovery methods for automotive lidar, software adaption was used the most and for a variety of faults (see Figure 8). Algorithms were used to mitigate security hacks, crosstalk, and filter out effects of unfavourable environmental conditions. In addition, software adaption methods were presented without going into detail about the fault, indicated as the “n/a” category in Figure 8. Hardware adaption was only used once to stabilize a lidar on a tractor [39]. Temperature adaption was used to recover from faults caused by a layer on the sensor cover and to keep the temperature within specifications. Naturally, cleaning of sensor cover recovery methods were applied solely when the fault was caused by an unwanted layer on the sensor cover.

**Figure 9:**
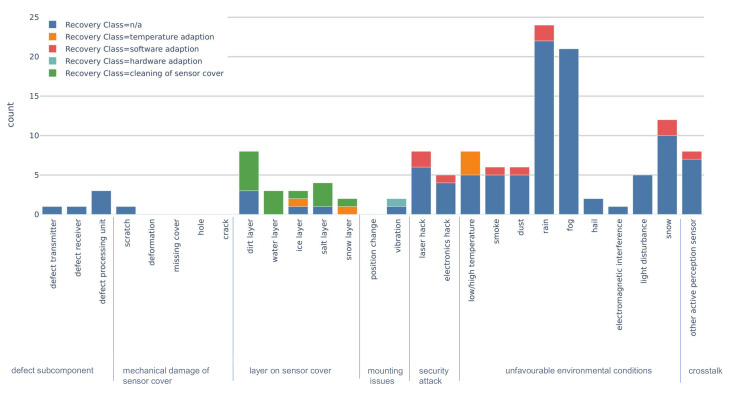
Exemplary faults (Table 1) and their associated recovery class count.

The recovery methods used for each individual exemplary fault shown in Table 1, are shown in Figure 9. The majority of publications, which discussed one or more faults, did not cover a suitable recovery method. Especially, faults of the class unfavourable environmental condition and defect subcomponent lacked suitable recovery methods.

Defect subcomponents are faults that are directly addressed by the manufacturer, and information is not readily available. Only some documentations of data protocols reveal hints about the internals. For example, in Ibeo [28], where error and warnings indicate internal fault detection.

Mechanical damage to the sensor cover by scratches was described in Rivero et al. [29] without a recovery method. However, cover related issues mostly dealt with a layer on the sensor cover.

Disruptive layers on the cover were mostly removed mechanically by air nozzles, spraying fluid, and wiping. Other publications approached the problem by shaking the unit [34] or by heating of the cover [36].

Only vibration was discussed as a mounting issue in the literature in Periu et al. [39]. They introduced an active stabilization mechanism to mitigate vibration effects on an agricultural vehicle. Changes of the sensor’s position were not covered in the literature.

Security attacks were addressed via prevention or software adaption. Dutta [47] developed an algorithm based on the least square estimator to mitigate attacks such as a denial of service and false data injection. Petit et al. [44] mitigated attacks with, besides redundancy and probing multiple times, algorithms based on increasing the difficulty for the hacker to hit the right time-window for attacks.

From the fault class unfavourable environmental condition rain and fog were mentioned the most (Figure 8). Nevertheless, only two publications [74,75] applied an algorithm to mitigate its negative effects. Both of them used a filter to minimize reflections from the particles in the air. Peynot et al. [75] use a Bayes filter and Zhang et al. [74] use a histogram and particle filter. Besides algorithms, temperature adaption methods are used to recover from environmental hazards. These are used to keep the sensor temperature within the specified range. Sick [86] used heating, while Wilson and Sinha [67] and Tegler [30] used cooling to keep the temperature within specifications.

Crosstalk by another automotive lidar was investigated by Kim et al. [17] and Zhang et al. [18]. They presented an adaptive algorithm to distinguish between target echo and crosstalk. There are also several patents, e.g., [89,91,92,93,94,117], which address methods for preventing crosstalk.

## 7. Discussion and Conclusions

### 7.1. Limitations of the Literature Study

Literature that might have used a different terminology could have been overlooked during the search, as well as methods buried deep in the text of patents. Furthermore, the interpretation of the classification of the FDII class is bound to some subjective judging. Nevertheless, the remaining 95 records have been read and interpreted carefully and analysed quantitatively.

### 7.2. Faults and Fault Classes

The presented lists of fault classes and, in particular, the exemplary faults are certainly not complete, but they are meant to cover all major issues which are of importance to reach the next step in terms of reliable environment perception. Out of our defined fault classes, unfavourable environmental conditions have been studied by far the most for lidar sensors, likely due to its frequent occurrence and its strong impact on the lidar perception principle.

In an alternative classification approach, faults could be classified according to the underlying physics. This would potentially allow common fault identification, isolation, and recovery methods per class. For example, the faults snow, dust, rain, smoke, and fog all can be described by scattering and multiple scattering. In more detail, the relevant description depends on the relationship between the particle size and the laser wavelength. Depending on the laser source, the wavelength is between 0.8 and 1.5 micrometers. Mie scattering can be used if the particle is of similar size compared to the wavelength, e.g., [118,119,120]. If the particle is smaller than the laser wavelength, Rayleigh scattering can be used. Otherwise, if the particle is much larger than the wavelength like it is the case for hail, non-selective scattering and absorption formulation is suitable. Scattering and absorption are not only relevant for precipitation, but also for issues related to the sensor cover.

### 7.3. Fault Detection, Isolation, and Identification

Fault detection, isolation, and identification methods based on monitoring the data of a single sensor are attractive since they are independent of other input sources. They can be directly implemented on the sensor firmware and are capable of detecting faults throughout all fault classes.

Third-party development of fault detection, isolation, and identification methods based on internal interfaces are hindered by the manufactures which are very protective of their intellectual property. This is especially the case for automotive lidar as the market is rather new, and competition is high.

### 7.4. Recovery Methods

Software adaption is the most commonly described recovery class and often implemented in the firmware of the sensor by the manufacturers. Algorithms for recovery can be applied directly to the raw data, before object detection.

### 7.5. Research Opportunities

Based on the presented survey, the following research opportunities can be identified:

- Mounting issues and mechanical damage to the sensor cover have been studied very little.
- Comparison to static and dynamic ground truth are very promising FDII methods. Still, these methods require more research to evaluate which and how ground truth information can be accessed by the FDIIR system.
- Although unfavourable environmental conditions have been discussed in many publications, possible recovery methods for this fault class were typically absent.
- Literature on defective subcomponents is sparse and no suitable recovery methods were described. This is most likely because commercial lidar units are provided as “black box”, because manufacturers are very protective of their intellectual properties. This calls for an open standard in order to access interfaces between subcomponents.
- Due to similar reasons, fault detection, isolation, and identification methods that access internal interfaces are mentioned only once in the literature.
- Detailed investigations of first principles of root causes for faults could lead to fault detection and recovery methods that apply for a wide range of faults.
- Hardware adaption has been studies very little and might be well suited for other faults related to mounting issues.

### 7.6. Closing Remarks

The introduced classification schema for faults, detection methods, and recovery methods, as well as the literature survey methodology and many of the presented fault detection and recovery methods, are not restricted to the automotive domain, but may also be applied to other domains such as aviation and maritime. The presented FDIIR classification and methods can be relevant for any automated system that requires a very reliable environment perception system (i.e., automated drones, automated trains, automated ships, automated airplanes, …).

In the last five years, an increasing interest in automotive perception sensors, especially lidar, and associated faults and recovery methods has been observed. However, there is still much to be done to offer reliable perception for ADAS/AD functions. Fault detection, isolation, identification, and recovery methods for automotive lidar are still at their infancy.

## Figures and Tables

**Table 1:** Classification of perception sensor faults including exemplary faults and related international safety standards.

Fault Class	Exemplary Fault	Safety Standard
defect subcomponent	defect transmitter	ISO 26262
	defect receiver	
	defect processing unit	
mechanical damage to sensor cover	deformation	ISO PAS 21448
	scratch	
	crack	
	hole	
	missing cover	
layer on sensor	dirt layer	
	water layer	
	ice layer	
	salt layer	
	snow layer	
mounting issue	position change	
	vibration	
security attack	laser hack	
	electronics hack	
unfavourable environmental condition	low/high temperature	
	dust	
	smoke	
	rain	
	fog	
	hail	
	electromagnetic interference	
	light disturbance	
	snow	
crosstalk	other active perception sensor	

**Table 2:** Classification of FDII methods for perception sensors including exemplary methods.

FDII Class	Exemplary Method
comparison to sensor model	objects detected by the sensor model compared to objects detected by the real sensor
monitoring sensor output	signal analysis and plausibility check of sensor output
comparison to static ground-truth	infrastructure detected by the sensor compared to ground-truth infrastructure in the environment
comparison to dynamic ground-truth	road users detected by another vehicle compared to road user detected by the ego-vehicle
comparison to other sensor of same type	compare objects that are detected by the sensor under observation with objects detected by another sensor of the same type (two lidar sensors)
comparison to other sensor of different type	compare objects that are detected by the sensor under observation with objects detected by another sensor of a different type (a lidar and a radar sensors)
monitoring internal interface	signal analysis and plausibility check of the output of a single sensor interface
comparison of multiple interfaces	a part of the sensor between sensor interfaces is modelled; comparison of the output of the modelled part with the output of the respective sensor interface

**Table 3:** Classification of fault recovery methods for perception sensors including exemplary methods.

Recovery Class	Exemplary Method
software adaption	filter pointcloud
	mitigate faults in sensor data by averaging
hardware adaption	change alignment of sensor cover
	adjust sensor position and rotation
temperature regulation	heating
	cooling
cleaning of sensor cover	air nozzle
	fluid nozzle
	wiper

**Table 4:** Literature review of fault classes, FDII and recovery methods for automotive lidar.

Fault Class	Literature
defect subcomponent	[26,27,28]
mechanical damage to sensor cover	[29]
layer on sensor cover	[29,30,31,32,33,34,35,36,37,38]
mounting issue	[39,40,41]
security attack	[15,16,42,43,44,45,46,47]
unfavourable environmental condition	[16,28,30,41,48,49,50,51,52,53,54,55,56,57,58,59,60,61,62,63,64,65,66,67,68,69,70,71,72,73,74,75,76,77,78,79,80,81,82,83,84,85,86,87,88]
crosstalk	[17,18,63,89,90,91,92,93,94]
**FDII Class**	
comparison to sensor model	[43,95,96]
monitoring sensor output	[18,26,33,38,41,43,46,47,53,60,84,90,97,98,99,100,101,102,103]
comparison to static ground-truth	[104,105]
comparison to dynamic ground-truth	[104,105,106,107]
comparison to other sensor of same type	[38,108,109,110]
comparison to other sensor of different type	[27,38,43,46,52,53,62,108,109,110,111,112]
monitoring internal interface	-
comparison of multiple interfaces	[113]
**Recovery Class**	
software adaption	[18,44,47,70,73,74,75,90,99,103,112,114,115,116]
hardware adaption	[39]
temperature adaption	[30,36,38,67,86]
cleaning of sensor cover	[30,32,34,35,37,38]

## Abbreviations

AD	Automated Driving
ADAS	Advanced Driver-Assistance Systems
ASIL	Automotive Safety Integrity Level
FDIIR	Fault Detection Isolation Identification Recovery
FOV	Field Of View
MEMS	MicroElectroMechanical Systems
SAE	Society of Automotive Engineers
V2V	Vehicle-to-vehicle communication
V2X	Vehicle-to-everything communication
